# *catena*-Poly[bis­(μ_3_-2-methyl-3,5-dinitro­benzoato)disilver(I)]

**DOI:** 10.1107/S1600536811022483

**Published:** 2011-06-18

**Authors:** Muhammad Danish, M. Nawaz Tahir, Sabiha Ghafoor, Nazir Ahmad, Mehwish Nisa

**Affiliations:** a Department of Chemistry, University of Gujrat, Hafiz Hayat Campus, Gujrat, Pakistan; b Department of Physics, University of Sargodha, Sargodha, Pakistan; c Department of Chemistry, University of Sargodha, Sargodha, Pakistan

## Abstract

In the title coordination polymer, [Ag_2_(C_8_H_5_N_2_O_6_)_2_]_*n*_, the silver ion is coordinated to three O atoms from three different anions in an approximate T-shape with one bond much longer than the other two. The polyhedral connectivity leads to [100] chains containing alternating centrosymmetric four-rings and eight-rings, with a short *d* ^10^⋯*d* ^10^ Ag⋯Ag interaction [2.8846 (4) Å] across the latter. The nitro groups are oriented at dihedral angles of 21.2 (5) and 64.3 (3)° with respect to the aromatic ring of the ligand. A C—H⋯O inter­action occurs in the crystal.

## Related literature

For background and related structures, see: Danish, Ghafoor, Ahmad *et al.* (2011 ▶); Danish, Ghafoor, Tahir *et al.* (2011 ▶); Danish, Tahir *et al.* (2011 ▶); Tahir *et al.* (1996 ▶, 2009 ▶); Ülkü *et al.* (1996 ▶). For graph-set notation, see: Bernstein *et al.* (1995 ▶).
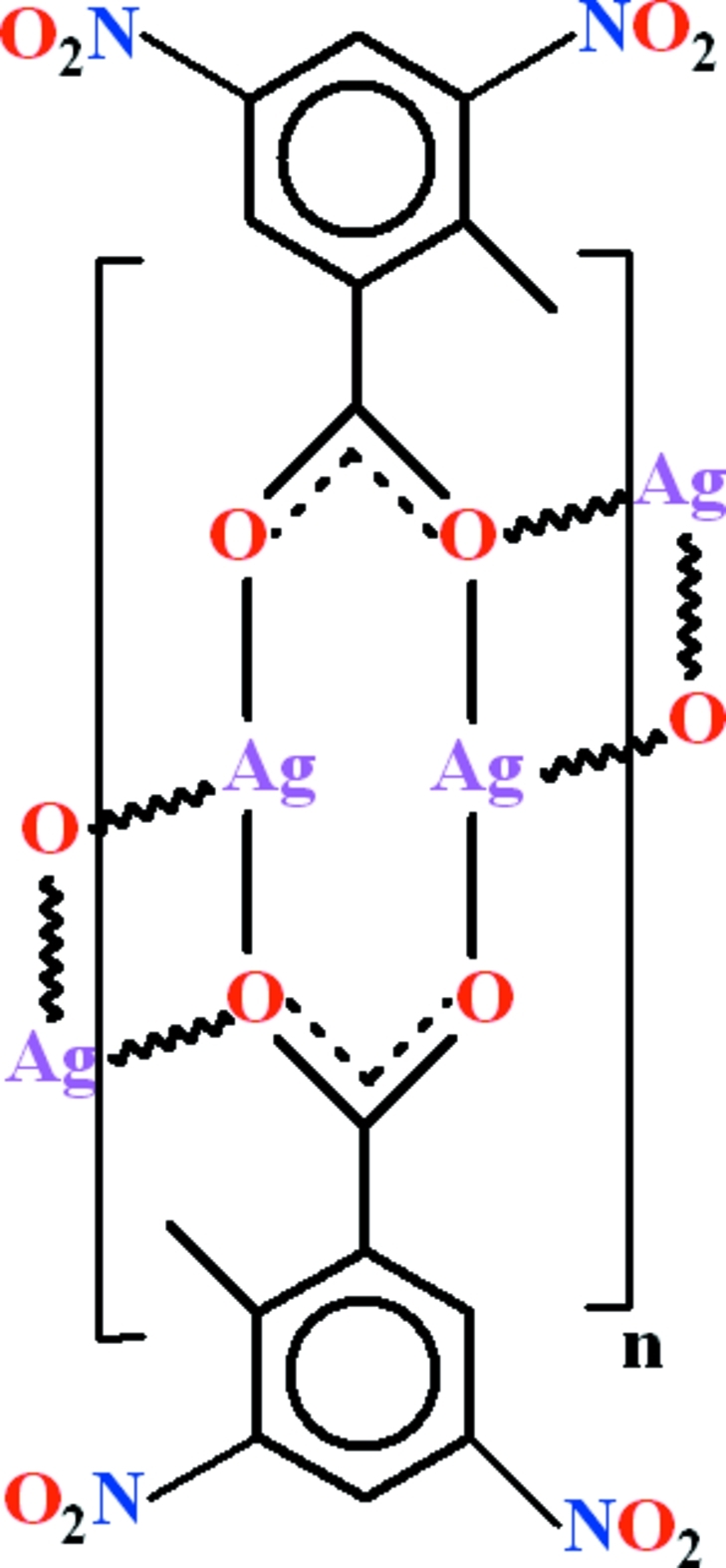


## Experimental

### 

#### Crystal data

[Ag_2_(C_8_H_5_N_2_O_6_)_2_]
*M* *_r_* = 333.01
Monoclinic, 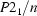

*a* = 5.7073 (3) Å
*b* = 11.9204 (6) Å
*c* = 14.5117 (7) Å
β = 90.493 (2)°
*V* = 987.24 (9) Å^3^
*Z* = 4
Mo *K*α radiation
μ = 2.06 mm^−1^
*T* = 296 K
0.32 × 0.24 × 0.22 mm

#### Data collection

Bruker Kappa APEXII CCD diffractometer
Absorption correction: multi-scan (*SADABS*; Bruker, 2005 ▶) *T*_min_ = 0.560, *T*_max_ = 0.630
9039 measured reflections
2406 independent reflections
1786 reflections with *I* > 2σ(*I*)
*R*_int_ = 0.037

#### Refinement

*R*[*F* ^2^ > 2σ(*F* ^2^)] = 0.031
*wR*(*F* ^2^) = 0.071
*S* = 1.02
2406 reflections
155 parameters
H-atom parameters constrained
Δρ_max_ = 0.37 e Å^−3^
Δρ_min_ = −0.47 e Å^−3^

### 

Data collection: *APEX2* (Bruker, 2009 ▶); cell refinement: *SAINT* (Bruker, 2009 ▶); data reduction: *SAINT*; program(s) used to solve structure: *SHELXS97* (Sheldrick, 2008 ▶); program(s) used to refine structure: *SHELXL97* (Sheldrick, 2008 ▶); molecular graphics: *ORTEP-3 for Windows* (Farrugia, 1997 ▶) and *PLATON* (Spek, 2009 ▶); software used to prepare material for publication: *WinGX* (Farrugia, 1999 ▶) and *PLATON*.

## Supplementary Material

Crystal structure: contains datablock(s) global, I. DOI: 10.1107/S1600536811022483/hb5898sup1.cif

Structure factors: contains datablock(s) I. DOI: 10.1107/S1600536811022483/hb5898Isup2.hkl

Additional supplementary materials:  crystallographic information; 3D view; checkCIF report

## Figures and Tables

**Table:** 

Ag1—O2^i^	2.190 (2)
Ag1—O1	2.227 (2)
Ag1—O1^ii^	2.502 (2)

**Table:** 

O2^i^—Ag1—O1	162.11 (8)
O2^i^—Ag1—O1^ii^	117.90 (8)
O1—Ag1—O1^ii^	76.52 (8)

Symmetry codes: (i) 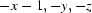; (ii) 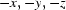.

**Table 2:** Hydrogen-bond geometry (Å, °)

*D*—H⋯*A*	*D*—H	H⋯*A*	*D*⋯*A*	*D*—H⋯*A*
C5—H5⋯O5^iii^	0.93	2.34	3.227 (4)	159

Symmetry code: (iii) 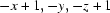.

**Fig. 1.**
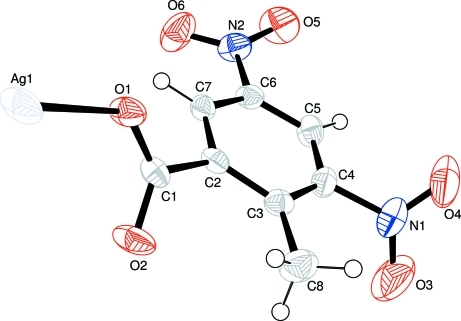
View of the asymmetric unit of (I) with displacement ellipsoids drawn at the 50% probability level.

**Fig. 2.**
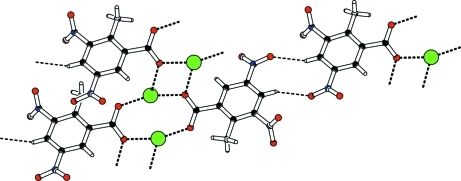
The partial packing of (I) showing the polymeric chains.

## supplementary crystallographic information

### Comment

The title compound (I, Fig. 1) is in continuation of the synthesis of metal complexes of 3,5-dinitro-*o*-toluic acid. We have reported the crystal structures of (II) *i.e.*, (methanol-κ*O*)(2-methyl-3,5-dinitrobenzoato-κ*O*)triphenyltin(IV) (Danish, Ghafoor, Ahmad *et al.*, 2011) and (III) *i.e.*, tetrakis(µ_2_-2-methyl-3,5-dinitrobenzoato-κ^2^*O*^1^:*O*^1'^)bis[aquacopper(II)] (Danish, Ghafoor, Tahir *et al.*, 2011).

We also reported the crystal structures of silver complexes such as (IV) *i.e.*, Poly[bis(*p*-nitrosalicylato-O:*O*^'^)disilver(I)—O^3^:Ag';Ag:*O*^3'^] (Tahir *et al.*, 1996), (V) *i.e.*, Poly[bis(3,5-dinitrobenzoato-O^1^:*O*^2^)disilver(I)—O^2^:Ag;Ag^'^:*O*^2'^] (Ülkü *et al.*, 1996), (VI) *i.e.*, Poly[(µ-benzene-1,2,4,5-tetracarboxylato)tetrasilver(I)] (Tahir *et al.*, 2009) and (VII) *catena*-Poly[bis(µ_3_-2-methylbenzoato) disilver(I)] (Danish, Tahir *et al.*, 2011).

In the title compound, toluene group A (C2—C8) is planar with r.m.s. deviation of 0.0139 Å. The silver-carboxylato group B (Ag1/O1/C1/O2) is also planar with r.m.s deviation of 0.0003 Å. The dihedral angle between A/B is 45.92 (11)°. The nitro groups C (O3/N1/O4) and D (O5/N2/O6) are of course planar. The dihedral angle between A/C, A/D and C/D is 64.26 (27), 21.22 (45) and 55.02 (37)°, respectively. The title compound consists of conventional centrosymmetric dimers with central core E (Ag1/O1/C1/O2/Ag1^i^/O2^i^/C1^i^/O1^i^: symmetry code i=-*x* - 1, -*y*, -*z*). There exist intermolecular H-bondings of C—H···O type (Table 2, Fig. 2) to form the *R*_2_^2^(10) ring motifs (Bernstein *et al.*, 1995). These chains are interlinked to form essentially three-dimensional polymeric chains. In the central core the range of Ag—O bond distances is [2.190 (2)–2.227 (2) Å] whereas the same for adjacent molecules is 2.502 (2) Å. The Ag···Ag distance for central core is 2.8846 (4) Å, whereas it is 3.7173 (4) Å for the symmetry related adjacent molecules forming four membered ring F (Ag_2_O_2_). The important bond distances and bond angles are given in Table 1.

### Experimental

Aqueous solutions of silver nitrate (0.17 g, 1.0 mmol) and the sodium salt of 2-methyl-3,5-dinitrobenzoic acid (0.248 g, 1.0 mmol) were prepared separately in 5.0 ml and 10.0 ml of water, respectively. The aqueous silver nitrate was dropwise added to the solution of sodium 2-methyl-3,5-dinitrobenzoate with continuous stirring till white precipitates were appeared. The reaction mixture was filtered after treatment with liquid ammonia. It was concentrated and kept for crystallization in dark. Colourless prisms of (I) appeared within two months.

### Refinement

The H-atoms were positioned geometrically (C–H = 0.93–0.96 Å) and refined as riding with *U*_iso_(H) = *xU*_eq_(C), where *x* = 1.5 for methyl and *x* = 1.2 for aryl H-atoms.

### Crystal data

**Table:**

[Ag_2_(C_8_H_5_N_2_O_6_)_2_]	*F*(000) = 648
*M**_r_* = 333.01	*D*_x_ = 2.240 Mg m^−^^3^
Monoclinic, *P*2_1_/*n*	Mo *K*α radiation, λ = 0.71073 Å
Hall symbol: -P 2yn	Cell parameters from 1786 reflections
*a* = 5.7073 (3) Å	θ = 2.2–28.3°
*b* = 11.9204 (6) Å	µ = 2.06 mm^−^^1^
*c* = 14.5117 (7) Å	*T* = 296 K
β = 90.493 (2)°	Prism, colorless
*V* = 987.24 (9) Å^3^	0.32 × 0.24 × 0.22 mm
*Z* = 4	

### Data collection

**Table:**

Bruker Kappa APEXII CCD diffractometer	2406 independent reflections
Radiation source: fine-focus sealed tube	1786 reflections with *I* > 2σ(*I*)
graphite	*R*_int_ = 0.037
Detector resolution: 7.50 pixels mm^-1^	θ_max_ = 28.3°, θ_min_ = 2.2°
ω scans	*h* = −7→7
Absorption correction: multi-scan (*SADABS*; Bruker, 2005)	*k* = −15→15
*T*_min_ = 0.560, *T*_max_ = 0.630	*l* = −19→18
9039 measured reflections	

### Refinement

**Table:**

Refinement on *F*^2^	Primary atom site location: structure-invariant direct methods
Least-squares matrix: full	Secondary atom site location: difference Fourier map
*R*[*F*^2^ > 2σ(*F*^2^)] = 0.031	Hydrogen site location: inferred from neighbouring sites
*wR*(*F*^2^) = 0.071	H-atom parameters constrained
*S* = 1.02	*w* = 1/[σ^2^(*F*_o_^2^) + (0.0305*P*)^2^ + 0.132*P*] where *P* = (*F*_o_^2^ + 2*F*_c_^2^)/3
2406 reflections	(Δ/σ)_max_ = 0.001
155 parameters	Δρ_max_ = 0.37 e Å^−^^3^
0 restraints	Δρ_min_ = −0.47 e Å^−^^3^

### Special details

**Table:**

Geometry. All e.s.d.'s (except the e.s.d. in the dihedral angle between two l.s. planes) are estimated using the full covariance matrix. The cell e.s.d.'s are taken into account individually in the estimation of e.s.d.'s in distances, angles and torsion angles; correlations between e.s.d.'s in cell parameters are only used when they are defined by crystal symmetry. An approximate (isotropic) treatment of cell e.s.d.'s is used for estimating e.s.d.'s involving l.s. planes.
Refinement. Refinement of *F*^2^ against ALL reflections. The weighted *R*-factor *wR* and goodness of fit *S* are based on *F*^2^, conventional *R*-factors *R* are based on *F*, with *F* set to zero for negative *F*^2^. The threshold expression of *F*^2^ > σ(*F*^2^) is used only for calculating *R*-factors(gt) *etc*. and is not relevant to the choice of reflections for refinement. *R*-factors based on *F*^2^ are statistically about twice as large as those based on *F*, and *R*- factors based on ALL data will be even larger.

### Fractional atomic coordinates and isotropic or equivalent isotropic displacement parameters (Å^2^)

**Table:**

	*x*	*y*	*z*	*U*_iso_*/*U*_eq_	
Ag1	−0.29331 (4)	−0.02958 (3)	−0.051068 (17)	0.04430 (11)	
O1	−0.1299 (4)	−0.0013 (2)	0.08689 (15)	0.0381 (6)	
O2	−0.4558 (4)	0.0300 (2)	0.16610 (16)	0.0459 (6)	
O3	−0.1538 (7)	0.2338 (3)	0.4925 (2)	0.0946 (12)	
O4	0.1858 (6)	0.2829 (3)	0.4489 (2)	0.0870 (11)	
O5	0.5695 (4)	−0.0999 (3)	0.40124 (18)	0.0609 (8)	
O6	0.3756 (5)	−0.2134 (2)	0.31332 (19)	0.0623 (8)	
N1	0.0174 (6)	0.2210 (2)	0.4468 (2)	0.0477 (8)	
N2	0.4018 (4)	−0.1237 (3)	0.35194 (18)	0.0400 (7)	
C1	−0.2392 (5)	0.0204 (2)	0.1589 (2)	0.0276 (6)	
C2	−0.0938 (5)	0.0338 (2)	0.24522 (19)	0.0252 (6)	
C3	−0.1291 (5)	0.1216 (2)	0.3078 (2)	0.0282 (6)	
C4	0.0286 (5)	0.1253 (3)	0.3821 (2)	0.0319 (7)	
C5	0.2037 (5)	0.0491 (3)	0.3986 (2)	0.0341 (7)	
H5	0.3050	0.0562	0.4488	0.041*	
C6	0.2220 (5)	−0.0381 (3)	0.3373 (2)	0.0289 (6)	
C7	0.0803 (5)	−0.0451 (2)	0.26043 (19)	0.0271 (6)	
H7	0.1016	−0.1031	0.2184	0.032*	
C8	−0.3140 (6)	0.2095 (3)	0.2935 (3)	0.0455 (9)	
H8A	−0.3469	0.2171	0.2288	0.068*	
H8B	−0.2592	0.2799	0.3175	0.068*	
H8C	−0.4541	0.1877	0.3249	0.068*	

### Atomic displacement parameters (Å^2^)

**Table:**

	*U*^11^	*U*^22^	*U*^33^	*U*^12^	*U*^13^	*U*^23^
Ag1	0.02496 (14)	0.0754 (2)	0.03242 (15)	−0.00299 (13)	−0.00920 (10)	0.00362 (13)
O1	0.0238 (10)	0.0648 (16)	0.0257 (12)	0.0004 (10)	−0.0030 (9)	−0.0048 (10)
O2	0.0195 (10)	0.0871 (19)	0.0311 (12)	−0.0002 (11)	−0.0038 (9)	−0.0001 (12)
O3	0.122 (3)	0.078 (2)	0.085 (3)	−0.005 (2)	0.052 (2)	−0.0341 (19)
O4	0.094 (2)	0.053 (2)	0.114 (3)	−0.0129 (17)	−0.012 (2)	−0.0364 (18)
O5	0.0420 (14)	0.088 (2)	0.0518 (16)	0.0154 (14)	−0.0241 (12)	−0.0037 (14)
O6	0.0634 (17)	0.0575 (18)	0.0657 (19)	0.0282 (14)	−0.0176 (14)	−0.0220 (14)
N1	0.067 (2)	0.0365 (17)	0.0391 (17)	0.0022 (17)	−0.0016 (16)	−0.0086 (13)
N2	0.0325 (14)	0.0564 (19)	0.0311 (15)	0.0101 (14)	−0.0058 (11)	−0.0007 (13)
C1	0.0239 (14)	0.0316 (16)	0.0271 (15)	−0.0053 (13)	−0.0045 (12)	0.0057 (12)
C2	0.0201 (13)	0.0339 (17)	0.0215 (14)	−0.0055 (13)	−0.0004 (11)	0.0034 (12)
C3	0.0272 (14)	0.0289 (16)	0.0285 (16)	−0.0013 (13)	0.0051 (12)	0.0043 (12)
C4	0.0389 (17)	0.0283 (16)	0.0286 (16)	−0.0047 (14)	0.0031 (13)	−0.0035 (12)
C5	0.0374 (17)	0.0390 (19)	0.0257 (16)	−0.0034 (14)	−0.0075 (13)	−0.0031 (12)
C6	0.0230 (14)	0.0376 (18)	0.0260 (15)	0.0014 (13)	−0.0012 (12)	−0.0004 (13)
C7	0.0252 (14)	0.0330 (18)	0.0230 (14)	−0.0047 (12)	0.0009 (12)	−0.0034 (11)
C8	0.0414 (18)	0.039 (2)	0.056 (2)	0.0103 (16)	0.0047 (17)	0.0006 (16)

### Geometric parameters (Å, °)

**Table:**

Ag1—O2^i^	2.190 (2)	C1—C2	1.505 (4)
Ag1—O1	2.227 (2)	C2—C7	1.384 (4)
Ag1—O1^ii^	2.502 (2)	C2—C3	1.402 (4)
Ag1—Ag1^i^	2.8845 (5)	C3—C4	1.399 (4)
O1—C1	1.249 (4)	C3—C8	1.500 (4)
O1—Ag1^ii^	2.502 (2)	C4—C5	1.370 (4)
O2—C1	1.247 (3)	C5—C6	1.373 (4)
O2—Ag1^i^	2.190 (2)	C5—H5	0.9300
O3—N1	1.195 (4)	C6—C7	1.375 (4)
O4—N1	1.212 (4)	C7—H7	0.9300
O5—N2	1.223 (3)	C8—H8A	0.9600
O6—N2	1.216 (4)	C8—H8B	0.9600
N1—C4	1.479 (4)	C8—H8C	0.9600
N2—C6	1.462 (4)		
			
O2^i^—Ag1—O1	162.11 (8)	C4—C3—C2	115.3 (3)
O2^i^—Ag1—O1^ii^	117.90 (8)	C4—C3—C8	122.1 (3)
O1—Ag1—O1^ii^	76.52 (8)	C2—C3—C8	122.5 (3)
O2^i^—Ag1—Ag1^i^	81.95 (6)	C5—C4—C3	125.3 (3)
O1—Ag1—Ag1^i^	80.74 (6)	C5—C4—N1	115.8 (3)
O1^ii^—Ag1—Ag1^i^	150.00 (5)	C3—C4—N1	118.8 (3)
C1—O1—Ag1	125.13 (18)	C4—C5—C6	116.6 (3)
C1—O1—Ag1^ii^	129.30 (18)	C4—C5—H5	121.7
Ag1—O1—Ag1^ii^	103.48 (8)	C6—C5—H5	121.7
C1—O2—Ag1^i^	125.3 (2)	C5—C6—C7	121.6 (3)
O3—N1—O4	124.0 (3)	C5—C6—N2	119.5 (3)
O3—N1—C4	119.4 (3)	C7—C6—N2	118.9 (3)
O4—N1—C4	116.6 (3)	C6—C7—C2	120.3 (3)
O6—N2—O5	124.5 (3)	C6—C7—H7	119.8
O6—N2—C6	117.6 (3)	C2—C7—H7	119.8
O5—N2—C6	117.9 (3)	C3—C8—H8A	109.5
O2—C1—O1	126.3 (3)	C3—C8—H8B	109.5
O2—C1—C2	117.4 (3)	H8A—C8—H8B	109.5
O1—C1—C2	116.3 (2)	C3—C8—H8C	109.5
C7—C2—C3	120.7 (3)	H8A—C8—H8C	109.5
C7—C2—C1	116.8 (3)	H8B—C8—H8C	109.5
C3—C2—C1	122.5 (3)		
			
O2^i^—Ag1—O1—C1	19.3 (5)	C2—C3—C4—C5	−2.3 (4)
O1^ii^—Ag1—O1—C1	164.8 (3)	C8—C3—C4—C5	−178.9 (3)
Ag1^i^—Ag1—O1—C1	4.5 (2)	C2—C3—C4—N1	174.6 (3)
O2^i^—Ag1—O1—Ag1^ii^	−145.5 (3)	C8—C3—C4—N1	−2.0 (4)
O1^ii^—Ag1—O1—Ag1^ii^	0.0	O3—N1—C4—C5	−118.4 (4)
Ag1^i^—Ag1—O1—Ag1^ii^	−160.29 (8)	O4—N1—C4—C5	62.5 (4)
Ag1^i^—O2—C1—O1	−7.0 (5)	O3—N1—C4—C3	64.4 (4)
Ag1^i^—O2—C1—C2	174.26 (19)	O4—N1—C4—C3	−114.7 (4)
Ag1—O1—C1—O2	−0.1 (5)	C3—C4—C5—C6	−0.8 (5)
Ag1^ii^—O1—C1—O2	160.7 (2)	N1—C4—C5—C6	−177.8 (3)
Ag1—O1—C1—C2	178.61 (18)	C4—C5—C6—C7	3.6 (5)
Ag1^ii^—O1—C1—C2	−20.6 (4)	C4—C5—C6—N2	−178.7 (3)
O2—C1—C2—C7	134.2 (3)	O6—N2—C6—C5	160.4 (3)
O1—C1—C2—C7	−44.6 (4)	O5—N2—C6—C5	−20.4 (4)
O2—C1—C2—C3	−46.2 (4)	O6—N2—C6—C7	−21.8 (4)
O1—C1—C2—C3	135.0 (3)	O5—N2—C6—C7	157.4 (3)
C7—C2—C3—C4	2.7 (4)	C5—C6—C7—C2	−3.2 (5)
C1—C2—C3—C4	−176.9 (3)	N2—C6—C7—C2	179.1 (3)
C7—C2—C3—C8	179.3 (3)	C3—C2—C7—C6	−0.2 (4)
C1—C2—C3—C8	−0.2 (4)	C1—C2—C7—C6	179.4 (3)

Symmetry codes: (i) −*x*−1, −*y*, −*z*; (ii) −*x*, −*y*, −*z*.

### Hydrogen-bond geometry (Å, °)

**Table:**

*D*—H···*A*	*D*—H	H···*A*	*D*···*A*	*D*—H···*A*
C5—H5···O5^iii^	0.93	2.34	3.227 (4)	159

Symmetry codes: (iii) −*x*+1, −*y*, −*z*+1.
